# The Concept of Optimal Dynamic Pedalling Rate and Its Application to Power Output and Fatigue in Track Cycling Sprinters—A Case Study

**DOI:** 10.3390/sports11010019

**Published:** 2023-01-16

**Authors:** Anna Katharina Dunst, Clemens Hesse, Olaf Ueberschär

**Affiliations:** 1 Department of Endurance Sports, Institute for Applied Training Science, Marschnerstraße 29, 04109 Leipzig, Germany; 2 Science Department, German Cycling Federation, 60528 Frankfurt/Main, Germany; 3 Department of Biomechanics, Institute for Applied Training Science, Marschnerstraße 29, 04109 Leipzig, Germany; 4 Department of Engineering and Industrial Design, Magdeburg-Stendal University of Applied Sciences, 39114 Magdeburg, Germany

**Keywords:** track cycling, optimisation, performance modelling, force–velocity profile

## Abstract

Sprint races in track cycling are characterised by maximal power requirements and high-power output over 15 to 75 s. As competition rules limit the athlete to a single gear, the choice of gear ratio has considerable impact on performance. Traditionally, a gear favouring short start times and rapid acceleration, i.e., lower transmission ratios, was chosen. In recent years, track cyclists tended to choose higher gear ratios instead. Based on a review of the relevant literature, we aimed to provide an explanation for that increase in the gear ratio chosen and apply this to a 1000 m time trial. Race data with continuous measurements of crank force and velocity of an elite track cyclist were analysed retrospectively regarding the influence of the selected gear on power, cadence and resulting speed. For this purpose, time-dependent maximal force-velocity (F/v) profiles were used to describe changes in performance with increasing fatigue. By applying these profiles to a physical model of track cycling, theoretical power output, cadence and resulting speed were calculated for different scenarios. Based on previous research results, we assume a systematic and predictable decline in optimal cadence with increasing fatigue. The choice of higher gear ratios seems to be explained physiologically by the successive reduction in optimal cadence as fatigue sets in. Our approach indicates that average power output can be significantly increased by selecting a gear ratio that minimises the difference between the realised cadence and the time-dependent dynamic optimum. In view of the additional effects of the gear selection on acceleration and speed, gear selection should optimally meet the various requirements of the respective sprint event.

## 1. Introduction

### 1.1. Background

Sprint track cycling races (i.e., team sprint, sprint, Keirin, and 1000 and 500 m time trials, as well as flying 200 m events) range from 15 s to 90 s in duration, depending on event, and require a maximal power output over distances of 250–1000 m [1].

Power requirements are physically linked to the choice of gear ratio, i.e., the ratio of chain ring teeth count relative to the number of teeth at the sprocket (e.g., 57/16), determining the deployment as propulsion per crank revolution (e.g., 7.48 m). This setup cannot be changed during the effort and must be chosen prior to a race. Depending on the setup and system weight, a specific inertial resistance has to be overcome at a certain cadence and system velocity. The gear ratio therefore augments the potential acceleration of the system [2]. As the acceleration period is a considerable part of sprint disciplines, traditionally a gear ratio in favour of short start- and acceleration times is chosen.

**Figure 1:**
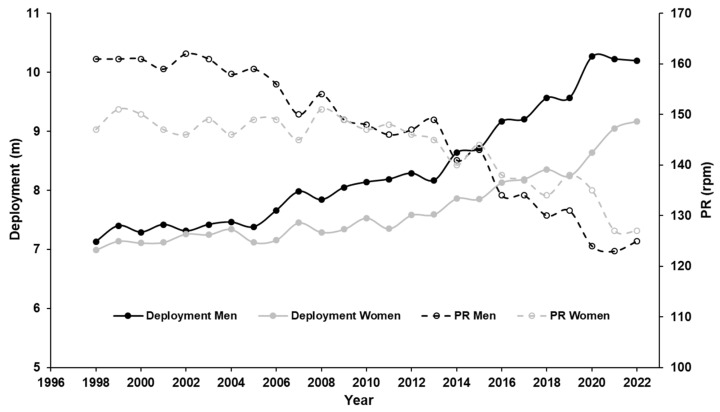
Development of gears by track cycling sprinters over the past 25 years, shown as deployment (i.e., distance cycled per revolution of the pedals), and resulting changes of mean pedalling rate. The values shown are the means for the 6 female and 10 male cyclists ranked highest in the flying 200 m at the world championships of the respective year.

In recent years, track cyclists tended to choose higher gear ratios, leading to higher resistance at the start and decreased mean pedalling rates [3]. In the men’s track cycling flying 200 m at the Olympic Games in 2000, the gear ratios chosen resulted in propulsion of less than 7.5 m per revolution, while in 2021 the corresponding value was 10 m or more. A similar tendency can be observed in female competitors. Figure 1 illustrates the changes in gear ratio and mean pedalling rates for the flying 200 m for elite female and male track cyclists over the past 25 years.

At the same time, a linear decrease in finishing time can be observed for both women (y = −0.0445 [s] ∗ x [yrs] + 11.662 [s], R^2^ = 0.860) and men (y = −0.0370 [s] ∗ x [yrs] + 10.500 [s], R^2^ = 0.888). Pearson’s correlation coefficient reveals a highly significant strong negative correlation between deployment and finishing time considering the past 25 years (r < −0.940, *p* < 0.001).

**Figure 2:**
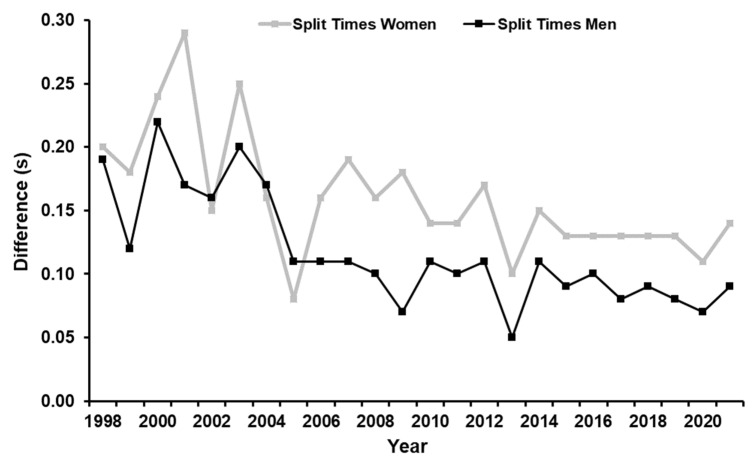
Development of the time difference of the split times for the first and second 100 m of the fastest 6 female and 10 male athletes in the flying 200 m race at the world championships from 1998 to 2022. Traditionally, the split time of the first half has been much faster than the split time of the second half. From 2005 onwards, there is a sudden decrease in the difference between the split times for the first and second 100 m, especially for men.

Particularly noteworthy is the altered time course of the flying 200 m with the years and thus, the gradually increasing gear ratios, as shown in Figure 2. Looking at the split times for the first and second 100 m in the flying 200 m race, there is a sudden decrease in the difference from 2005 onwards, indicating a possible advantage of higher gear ratios with increasing distance and duration of the effort.

### 1.2. The Relationship between Force–Velocity and Power–Velocity in Track Cycling

A decisive physiological determinant of performance in track cycling sprints is the neuro-muscular capacity, i.e., the ability to produce high fatigue-free power output, which can be described by maximal force–velocity (F/v) and power–velocity (P/v) profiles [1,4,5,6]. Hill [7] modelled the velocity of the shortening of single muscle fibres as a rectangular hyperbola, but in multi-joint movements with resistance a strong linear relation between the force generated and velocity of movement has been established as standard model [8,9]. In cycling, the F/v relationship of mean pedal force and pedalling rate (PR) was identified as linear and, consequently, the relationship between power output (P) and pedalling rate as parabolic. Both can be derived from short maximal sprints in laboratory and in field [5,6,10,11,12,13,14].

In track cycling, valid maximal F/v and P/v profiles can be derived from fatigue-free maximal accelerations with sufficiently wide cadence spectra [6]. The functions established can be used to extra- and interpolate parameters as the current maximal mean pedal force (F_max_)*,* maximal pedalling rate (PR_max_), maximal power output (P_max_) and current optimal pedalling rate (PR_opt_) corresponding to P_max_ [5,6,15].

### 1.3. The Force–Velocity Relationship and Fatigue

With increasing duration of work, an athlete’s metabolic performance (as reflected in resistance to fatigue) becomes increasingly important as it determines the decay of the maximal power output after the onset of fatigue [2,3,16]. In all-out phases during sprint races, where mechanical power exerted by the athlete is equal to the maximal power attainable for the present pedal rate and exhaustion state, power output decreases exponentially due to fatigue [1,16]. To maximise mean power output, the power output (during such specific training sessions) should always be equal to the maximal power that is physiologically available for the current cadence and state of exhaustion [16]. Due to the parabolic relationship of power and pedalling rate and the associated differences in muscle fibres recruitment patterns [17], the fatigue-induced drop in power depends on cadence [18]. To control the effects of cadence on power decay during maximal sprints, an isokinetic test design is often used in anaerobic power diagnostics [19] as power decay and the biomechanical effect of cadence have not been differentiated so far.

Buttelli and colleagues [20] investigated the effect of increasing fatigue resulting from four consecutive short maximal voluntary accelerations on a bicycle ergometer without rest on the F/v relationship. They found a parallel down shift of the current F/v profile from the fatigue-free baseline towards lower velocities, corresponding to a linear reduction in velocity with constant resistance. Similar patterns were found in animal experiments with rats [17], and validated in various isolated and complex movements in humans [21,22,23]. The maximal power output depends on velocity and changes systematically and predictably with the athlete’s resistance to fatigue. If the slope of the (maximum voluntary) F/v profile remains constant at any point on the load curve until the myosin heavy chain (MHC) IIx muscle fibres are fatigued [1], F/v and P/v profiles reflect the current work capacity. A change of the slope is to be expected when MHC IIx fibres are completely fatigued, as the F/v and P/v profiles of the more slowly contracting MHC isoforms differ [17,24]. With a parallel shift of the F/v profile towards the origin, the maximal and optimal pedalling rates decline in a predictable fashion, so that the optimal cadence in exhaustive exercise is determined by fatigue [16].

Dorel and co-workers [5] hypothesised that even slight differences between the optimal and average pedalling rates $\left({\mathrm{PR}}_{\mathrm{mean}}\right)$ might significantly impact average power output during a race ($P_{\mathrm{mean}})$, but neglected fatigue-induced changes in their calculations. Our recent results show an exponential decrease of optimal cadence in maximal sprints with increasing duration (dynamic optimal pedalling rate) and indicate that applying duration-dependent mean optimal pedalling rates maximises average power output in an isokinetic mode [16].

In the following, we apply our recently described mathematical approach to 1000 m time trial data of a world-class track cyclists of the German national track cycling team to illustrate fatigue-induced changes of the optimal pedalling rate and aim to demonstrate the physiological benefits of higher gear ratios.

The specific demands of the 1000 m time trial, consisting of a standing start and, usually, an all-out effort over the entire duration [1], allow for modelling of neuro-muscular capacity (i.e., maximal crank force and maximal crank velocity), resistance to fatigue and endurance, i.e., aerobic and glycolytic capacity, as well as resisting forces such as mass, aerodynamic and rolling resistances and drivetrain losses [2]. As performance in the flying 200 m derives from the same limiting capacities and resistances, it represents another use case of our model, which is, however, much more difficult to describe formally due to more complex degrees of freedom. Nevertheless, the physiological effect behind the higher gear ratios can be adequately investigated in a technical way by considering the 1000 m time trial. The findings can then be transferred to the flying 200 m application (see Discussion).

## 2. Methods

### 2.1. Test Design

An elite track cyclist (age: 22 years; height: 1.92 m; weight: 96 kg; BFP: 10%; P_max_: 2040 W; $\dot{V}$O_2max_: 63 mL kg^−1^ min^−1^) performed a 1000 m time trial in an indoor velodrome with a wooden surface of 250 m as part of an official track cycling sprint event. He followed an all-out fashion pacing, i.e., started as hard as possible and continued with the hardest effort voluntarily achievable. The gear ratio chosen was 3.87:1 (58/15), corresponding to a deployment of 8.12 m. Pedal force and crank velocity were measured continuously during the warm-up and the race with a power meter (FES, Institute for Research and Development of Sports Equipment, Berlin, Germany) recording the tangential force on the crank with a sampling frequency of 200 Hz and the duration of each crank revolution. This system allows for the creation of sport-specific F/v and P/v profiles [6]. Raw data was exported and further processed in Office Excel 2016 (Microsoft Corporation, Redmond, WA, USA). Based on the raw data of time-dependent power output, pedalling rate and resulting speed were calculated using our recent published mathematical approach [2,16].

As the time trial was part of a competition lasting several days, an individualized nutrition plan was followed, designed to optimize performance in and recovery in between the races. The focus was to maintain blood-sugar levels and avoid depletion of glycogen stores in liver and the muscle.

The athlete retrospectively provided his written consent to use his data in this study, which was approved by the institute’s ethical committee and performed in accordance with the Declaration of Helsinki.

### 2.2. Data Analysis

**Figure 3:**
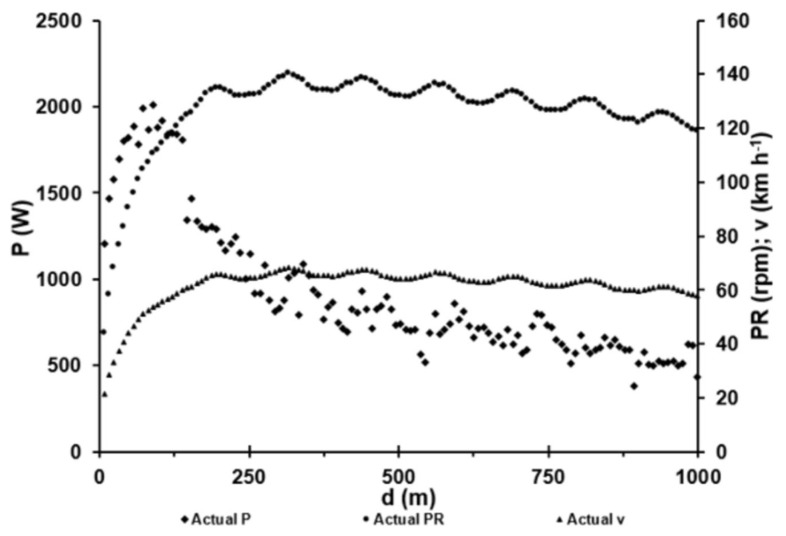
Mean power output (P) for each revolution of the crank (black squares) and the corresponding pedalling rates (PR; black dots) and speeds (black triangles) in a 1000 m time trial by an elite track cyclist. The gear ratio of 3.87:1 (58/15) corresponded to a deployment of 8.12 m. In the race, the mean power was 918 W at a mean cadence of 126 rpm. The oscillating data is induced by the design of the racing velodrome.

In track cycling time trials, athletes start from a standing position into the initial phase (acceleration phase) and switch to a seated position at a higher speed, usually before reaching the second curve [1]. In the 1000 m time trial of the present study, the athlete accelerated maximally from a stationary start in standing position and sat down after 12 s at ~145 m. He then completed the race in a seated position. The average pedalling rate was 126 rpm, with an average power output of 918 W. Figure 3 shows the power output, pedalling rate and speed.

Previous studies have shown that cycling in a standing position can increase power output by 8–12%, which has been attributed to additional power development from the upper body [25,26,27,28]. To accurately analyse the race data and to calculate the fatigue-induced changes in optimal cadence, separate fatigue-free F/v and P/v profiles were created for a standing and a seated position on the bike, as described in a previous publication [6].

As part of his warm-up program, the athlete performed 6 s of maximally high-cadence low-resistance cycling on a free roller (motoric sprint) and a short maximal sprint in a seated position on the track. The first 4 revolutions of the acceleration phase during this sprint and, as suggested by Dunst et al. [6], 1 or 2 revolutions with pedalling rates above 160 rpm derived from the first 3 s of the motoric sprint were used to create the current fatigue-free F/v and P/v profiles in the seated position. The data points with the best F/v relationship were selected.

To reflect the maximal mechanical performance of the athlete in the standing position on the bike, the F/v and P/v profiles were adjusted for the first 4 cycles of the acceleration phase during the standing start of the race [10,13]. The profile parameters were determined by linear and non-linear regression analysis and the theoretical maximal mean crank force F_max_, the theoretical maximal pedalling rate PR_max_ and the maximal power output P_max_ at optimal pedalling rate PR_opt_ were derived.

## 3. Results

**Figure 4:**
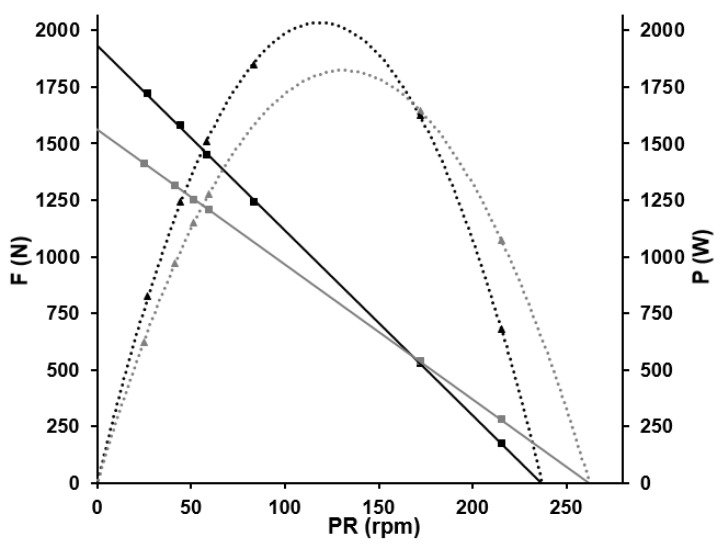
Fatigue-free force–velocity profiles and power–velocity profiles of the athlete in standing (black) and seated positions (grey) calculated (black and grey squares) by linear and non-linear regression analysis. In standing position, F_max_ was 1932 N and PR_max_ amounted to 237 rpm with a corresponding slope of −8.15 N rpm^−1^. In seated position, calculations yielded F_max_ = 1561 N, PR_max_ = 262 rpm and a slope of −5.95 N rpm^−1^.

For the standing position, theoretical maximal values of 1932 N and 237 rpm for force and pedalling rate (corresponding to a slope of −8.15 N rpm^−1^) and a maximal power output of 2040 W at an optimal cadence of 119 rpm were obtained. In a seated position, fatigue-free maximal mean pedal force was 1561 N and maximal cadence 262 rpm with a maximal power output of 1822 W at an optimal cadence of 131 rpm. The slope of the F/v profile in seated position was −5.95 N rpm^−1^. In all regressions, the coefficient of determination was R^2^ > 0.99 and maximal power output in the standing position was 12% higher than in the seated position. Figure 4 shows the fatigue-free F/v profiles and P/v profiles of the athlete in standing (black) and seated position (grey).

As the F/v profile shifts away from the fatigue-free profile towards the origin in a parallel manner with the onset of fatigue, F/v and P/v profiles for every crank revolution can be calculated from the current pedalling rate and corresponding mean pedal force while maintaining the slope of the fatigue-free F/v profile [16]. By substituting the time-dependent optimal cadence for the time-dependent state of the F/v and P/v profiles, the time-dependent maximal power output for each crank revolution can be determined (ibid.).

**Figure 5:**
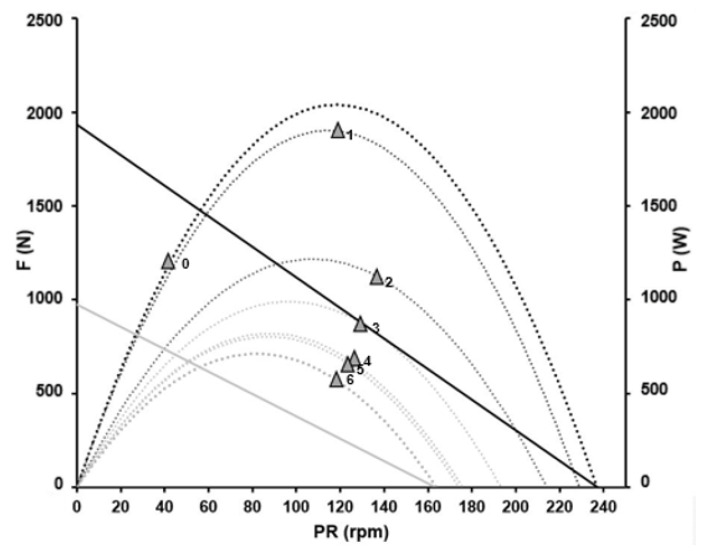
The F/v and P/v profiles of an elite track cyclist in the absence of fatigue (straight black line, standing position) and at the end of a 1000 m time trial (grey straight line, seated position). During the race, the maximal mean pedal force (F_max_) decreased from 1932 N to 998 N, the maximal power output (P_max_) from 2040 W to 745 W and the optimal pedalling rate (PR_opt_) declined from 119 rpm (standing position) or 131 rpm (seated position) to 84 rpm. The triangles represent the raw data points of the current pedalling rate with corresponding power output every 10th second from the first to the last pedal revolution in chronological order.

The F/v profile at the beginning (black straight line, standing position) and at the end (grey straight line, seated position) of the race as well as the P/v profiles every 10th second (dotted lines) are depicted in Figure 5. The profiles were derived from the last crank revolution of every 10th second interval. The triangles show the realised pedalling rate and power output.

## 4. Discussion

According to this approach, the optimal pedalling rate as given by the apex of the current P/v profile is not a static parameter, but a dynamic characteristic linked to fatigue. Its main factors are the athlete’s fatigue-free performance at the beginning of the race, their resistance to fatigue in terms of the rate of performance degradation and current position on the bike [3,16]. During the race, the athlete’s realised cadence deviated from the time-dependent optimum most of the time. As mentioned above, minimising the difference between a cyclist’s optimal and mean cadences can maximise mean power output. Considering only the time-dependent biomechanical optimum, the average optimal pedalling rate is 97 rpm, with a corresponding average power output of 1042 W or 114% of the realised value in an isokinetic effort.

**Figure 6:**
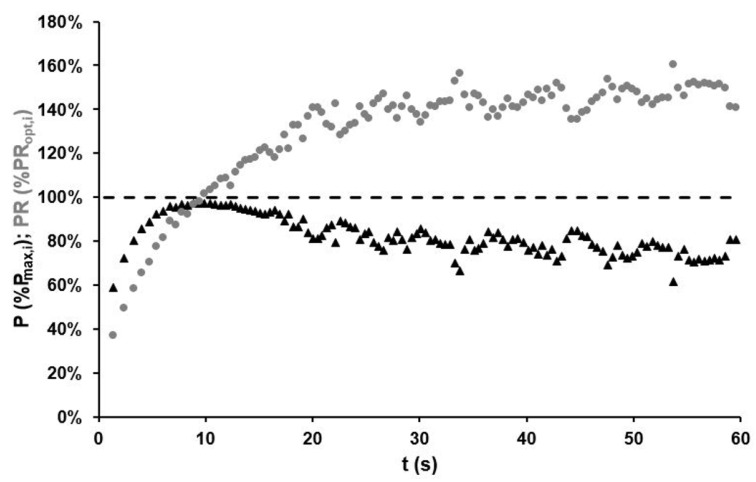
Comparison of actual pedalling rate PR_i_ (grey dots) and actual power output P_i_ (black triangles) as percentage of the dynamic optimum of pedalling rate PR_opt,i_ and dynamic maximal power output P_max,i_ for each crank revolution i at its corresponding time t during the 1000 m time trial.

An isokinetic setting would fix the cadence, also neglecting the fatigue-dependent drop in optimal cadence in a maximal effort. If the cadence at every revolution could be matched to the fatigue-dependent optimum, the average power output could be raised to 1190 W or 130% of the actual mean value. Figure 6 shows the theoretical potential for increased power output, especially in the initial acceleration and later phase of the 1000-m time trial.

The selection of higher gear ratios by elite track cyclists could therefore be explained by an endeavour (be it intentional or subconscious) to increase the mean power output during efforts. The extent of the effect, as shown in Figure 6, depends on the performance characteristics of the athlete and the duration of the effort. The lower the athlete’s maximal pedalling rate is, the lower their resistance to fatigue and the longer the race distance, the greater the positive effect of higher gear ratios will be on mean power output.

Maximising the highest or average power seems to be particularly appropriate in the context of optimal training stimuli. Considering the various resistances acting on rider and bike during a race, the goal could be to maximising either acceleration or (average) speed, depending on event and race strategy [2].

In events with a stationary start the performance reserves observed during the first 10 s of the time trial, where the actually achieved power output, is on average ~30% lower than the optimum (see Figure 6). These may only be tapped by selecting a relatively small gear ratio, which allows a very rapid increase in cadence. However, such a choice would decrease the potential power output after only a few seconds, as approaching very high cadences far above the fatigue-free optimum would counteract the fatigue-induced decrease in optimal cadence over time.

**Figure 7:**
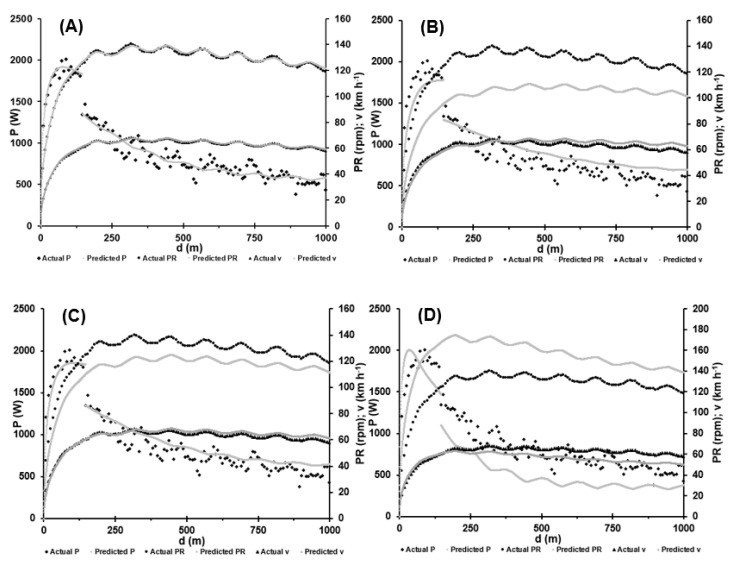
Pedalling rate (PR), power output (P) and speed (v) calculated for a gear ratio of (**A**) 3.87:1 (58/15) to rebuild the actual race, (**B**) 4.92:1 (59/12) to maximise mean power output, (**C**) 4.36:1 (61/14) to minimise finishing time over 1000 m and (**D**) 2.88:1 (49/17) to minimise finishing time over 250 m. Time-dependent power output, pedalling rates and resulting speeds were estimated applying our recently published mathematical approach to the raw data [2].

In contrast, larger gear ratios cause a reduction of potential power output during the initial acceleration phase due to the greater (inertial) resistance. If the effort is not dominated by the acceleration phase, the disadvantage of a large gear ratio can be compensated for by the subsequent higher power output. To demonstrate the impact of gear ratio on pedalling rate, power output and speed, the optimal gear ratios for three different scenarios were calculated while maintaining the environmental conditions of the race and are visualized in Figure 7. The original run (A) and optimizations for maximal average power output (B), minimal finishing time over 1000 m (C) and minimal fishing time over 250 m (D).

In the flying 200 m race, the rider enters the track with 3.5 laps to go and successively gains height on the bank during the first lap. Depending on the individual race tactics (athlete’s performance profile, selected gear ratio and associated acceleration distance), the first 1 to 1.5 laps (preparation phase) are run at relatively low speed (30–40 km h^−1^) and with the least effort possible. In the final 1–1.5 laps prior time measurement, the rider usually accelerates sharply, riding the at the top of the wooden track. Utilizing the potential energy, the rider accelerates from the highest point of the bank towards the measuring line that indicates the shortest legal track, to enter the 200 m distance at a very high speed. The aim is to complete the 200 m distance in the shortest possible time. Although the measured 200 m times of world-class female and male track cyclists are less than 10.5 s and 9.5 s, respectively, the total time of high to maximal effort is about 20 to 30 s. The preparatory phase of the first 1-1.5 laps lasts approximately one minute and can significantly contribute to fatigue before the start of the acceleration phase, depending on the endurance capacity and individual pacing (own unpublished research). This corresponds to fatigue after approx. 15 s of maximal effort, so that the level of the optimal pedalling rate during the actual 200 m distance corresponds to that after approximately 30 s of an all-out effort.

## 5. Practical Applications

The demands of track cycling sprint events can be described by the change in F/v profile over the time course of an effort due to fatigue. A key finding of our approach is that average power output can be significantly increased by choosing a gear ratio that minimises the difference between the actual and dynamically optimal pedalling rate. From a physiological perspective, the increased gear ratios required to compete at a cadence close to the optimal pedalling rate during the race lead to an increased demand for maximal strength and maximal power, especially during the acceleration phase. In sprint disciplines, an increase in maximal strength and power levels should always be achieved while maintaining the maximal sport-specific movement velocity, which is represented by the maximal pedalling rate, as a reduction could lead to a decrease in the fatigue-free optimal pedalling rate even before the start of a race [2].

It remains to be discussed whether the increased neuro-muscular demand can be adequately counterbalanced by increasing an athlete’s resistance to fatigue so that the drop in the optimal pedalling rate over time diminishes. In agreement with Douglas et al. [1], our research results indicate that a higher fatigue-free neuro-muscular performance outweighs an increased resistance to fatigue because of the computationally larger impact on the power integral [2]. The trend towards higher gear ratios consequently leads to a shift in the sport-specific physiological requirement profile towards a higher impact of the fatigue-free performance level and a decreased value of the resistance to fatigue in track cycling sprint events.

## 6. Conclusions

The trend towards the selection of higher gear ratios, and thus lower pedalling rates, by elite track cycling sprinters may be explained physiologically by the reduction in optimal cadence due to the onset of fatigue. Average power output can be increased significantly by choosing a gear ratio that minimises the difference between the actual and dynamic optimal pedalling rate. However, it must be kept in mind that increasing average power by choosing a higher gear ratio may be detrimental to competition result if the impact of higher gear ratios on the inertial loads and resistive forces to be overcome by the cyclist are not considered.

## Data Availability

The datasets generated during and/or analysed during the current study can be obtained from the corresponding author upon reasonable request.

## Abbreviations

a	Slope of the F/v profile
b	*y*-axis intercept of the F/v profile
b_i_	*y*-axis intercept of the F/v profile at crank revolution i
BFP	Body Fat Percentage
d	Distance
F/v	Force-velocity
F	Mean pedal force
F_max_	Maximal mean pedal force
F_max,i_	Maximal mean pedal force at crank revolution i
i	Number of crank revolutions; i = 1, 2, …
MHC	Myosin heavy chain
P	Power
P_max_	Maximum power output
P_max,i_	Maximal power output at crank revolution i
P_mean_	Average power output
PR	Pedalling rate, cadence, frequency
PR_max_	Maximal pedalling rate
PR_opt_	Optimal pedalling rate
PR_opt,i_	Optimal pedalling rate at crank revolution i
PR_opt_(t)	Optimal dynamic pedalling rate at timepoint t; time-dependent optimal pedalling rate
P/v	Power-velocity
rpm	Revolutions per minute
v	Velocity
v_max_	Maximal velocity
